# Programmed death receptor ligand-2 (PD-L2) bearing extracellular vesicles as a new biomarker to identify early triple-negative breast cancer patients at high risk for relapse

**DOI:** 10.1007/s00432-022-03980-9

**Published:** 2022-04-02

**Authors:** Oliver Hoffmann, Sebastian Wormland, Ann-Kathrin Bittner, Monika Collenburg, Peter A. Horn, Rainer Kimmig, Sabine Kasimir-Bauer, Vera Rebmann

**Affiliations:** 1 Department of Gynecology and Obstetrics, University Hospital of Essen, Hufelandstrasse 55, 45122 Essen, Germany; 2 Institute for Transfusion Medicine, University Hospital Essen, Hufelandstrasse 55, 45122 Essen, Germany

**Keywords:** Triple-negative breast cancer, Extracellular vesicles, PD-L2, PD-L1, PD-1, CTC

## Abstract

**Purpose:**

Based on the tumor-promoting features of extracellular vesicles (EV) and PD-L1/2-bearing EV subpopulations (PD-L1/2_EV_), we evaluated their potential as surrogate markers for disease progression or eligibility criteria for PD-1 immune checkpoint inhibition (ICI) approaches in early triple-negative breast cancer (TNBC).

**Methods:**

After enrichment of EV from plasma samples of 56 patients before and 50 after chemotherapy (CT), we determined levels of EV particle number and PD-L1/2_EV_ by nanoparticle tracking analysis or ELISA and associated the results with clinical status/outcome and the presence of distinct circulating tumor cells (CTC) subpopulations.

**Results:**

Compared to healthy controls, patients had a tenfold higher EV concentration and significantly elevated PD L2_EV_ but not PD L1_EV_ levels. The most important clinical implications were found for PD-L2_EV_. High PD-L2_EV_ levels were associated with a significantly reduced 3-year progression-free and overall survival (PFS and OS). A loss of PD-L2_EV_ after CT was significantly more prominent in patients achieving pathological complete response (pCR). Increased pre-CT PD-L2_EV_ levels were found in patients having NOTCH1-positive or ERBB3-positive CTC. The presence of ERBB3-positive CTC combined with high pre-CT PD-L2_EV_ resulted in a shorter PFS.

**Conclusion:**

This study highlights PD L2_EV_ as a promising biomarker for risk assessment of TNBC patients and represents the basic for additional studies introducing PD-L2_EV_ as an eligibility criterion for PD-1 ICI approaches.

**Supplementary Information:**

The online version contains supplementary material available at 10.1007/s00432-022-03980-9.

## Background

Breast cancer (BC) is the most common cancer in women worldwide with almost 2.1 million new diagnoses in 2018 (Bray et al. 2018; Sung et al. 2021). Although the 5-year overall survival (OS) rate is about 90% (Rose and Puckett 2021), the subgroup of triple-negative breast cancer (TNBC) patients, accounting for 15–20% of the cases, shows an aggressive behavior, which is associated with poor prognosis (Sharma 2018; Schneeweiss et al. 2019). TNBC is defined by the lack of estrogen and progesterone receptor expression and by the absence of human epidermal growth factor receptor 2 (HER2) overexpression. Up till now, neoadjuvant chemotherapy (NACT) remains to be the standard of care for TNBC (Isakoff 2010; Cardoso et al. 2019).

However, despite achieving a pathological complete response (pCR), a surrogate marker for improved progression-free survival (PFS) and OS, about 5–20% of patients eventually experience relapse (Biswas et al. 2017). Treatment options are limited since TNBC remains a biologically variable disease (Khosravi-Shahi et al. 2019). To improve outcome in TNBC, targeted immunotherapeutic approaches are currently under investigation (Schmid et al. 2020a, b, c; Manjunath and Choudhary 2021). Among them, the interaction of the programmed death receptor-1 (PD-1) with its cognate ligands PD-L1 or PD-L2 represents a crucial pathway to evade immune recognition in all kinds of tumor entities including TNBC. Indeed, immune checkpoint inhibition (ICI) by blocking PD-1 (Bian et al. 2019; Schmid et al. 2019) or its cognate ligand PD-L1 (Sabatier et al. 2015; Heimes and Schmidt 2019) in combination with NACT has the potential to substantially improve disease outcome in TNBC. A significant improvement in the pCR rate and in event-free survival (EFS) as well as a trend for improved OS has been shown in this setting (Schmid et al. 2021). The primary endpoint of improved pCR was met by the addition of Atezolizumab, a PD-L1 inhibitor, to NACT in the IMpassion031-trial (Mittendorf et al. 2020). Recently, it could be demonstrated that patients achieving a pCR after NACT in combination with Durvalumab (PD-1-Inhibitor) had a better OS compared to those achieving a pCR receiving no immunotherapy in the GeparNUEVO trial (Loibl et al. 2021). Interestingly, in contrast to the metastastic setting, a clinical benefit was seen independently of the expression of PD-L1 on tumor cells in the early setting of TNBC (Cortes et al. 2020; Mittendorf et al. 2020; Schmid et al. 2020a, b, c; Schmid et al. 2021). Consequently, a concomitant development of possible biomarkers is required (i) to identify patients with high risk of recurrence in the early setting or (ii) to select patients benefiting from PD-1 ICI or other targeted immunotherapies.

Since access to tumor tissue is limited, the use of blood as a liquid biopsy, comprising circulating tumor cells (CTC) and extracellular vesicles (EV) with their subpopulations represents a promising platform to establish surrogate markers in TNBC. The molecular characterization of CTC mRNA profiling has already identified certain combinations of CTC subpopulations being associated with outcome in primary TNBC (Bittner et al. 2020). EVs are of translational interest for tumor monitoring and prediction of therapy response, as nearly all cells, including BC cells, release EV into the circulation, and the cellular source of EV guides their molecular composition and cargo of bioactive effector molecules (cytokines, transcription factors, growth factors, oncogenic proteins, and genetic information, such as mRNA, microRNA proteins, lipids and nucleic acids). EVs are functionally operative in cell–cell communication by transferring their content information to adjacent or distant recipient cells (Gyorgy et al. 2011; Yanez-Mo et al. 2015; Konig et al. 2017), thereby, being able to contribute to pathways of tumor immune escape, tumor initiating, growth, spreading, and therapy resistance (Mashouri et al. 2019). Concerning the PD-1/PD-L1 axis, PD-L1-bearing EV (PD-L1_EV_) derived from BC has the capacity to transfer functional active PD-L1 to other cells and to interact with PD-1 receptor, which results in the inhibition of T cell activation as well as T cell killing of BC cells (Yang et al. 2018). Although PD-L2 is expressed in a variety of immune, stromal and BC cells (Baptista et al. 2016; Asano et al. 2018) and displays up to six-fold higher affinity to PD-1 than PD-L1 (Keir et al. 2008), PD-L2-bearing EVs (PD-L2_EV_) have attracted less interest as a biomarker in BC or other tumor entities.

Despite the established tumor-promoting role of EV within the tumor microenvironment, it has not been clarified at all, whether EVs derived from liquid biopsies of the blood and/or their subpopulations of PD-L1 and PD-L2 are meaningful surrogate marker(s) for disease progression or potential meaningful selection element(s) for PD-1 ICI therapy approaches in TNBC. To address these issues, we enriched circulating EV from 106 plasma samples procured before (*n* = 56) or after (*n* = 50) chemotherapy (CT) from 64 primary TNBC patients. EV particle concentrations and levels of PD-L1/2_EV_ subpopulations were analyzed in association with (i) routinely determined clinical parameters, (ii) the presence of distinct CTC subpopulations, and (iii) their prognostic importance in terms of PFS and OS.

## Materials and methods

### Patient population and patient characteristics

This retrospective study was conducted at the Department of Gynecology and Obstetrics, University Hospital of Essen, Germany. 64 TNBC patients (56 pre-CT, and 50 post-CT; 42 paired patients) diagnosed between January 2013 and August 2018, were enrolled in this study (Table 1).

**Table 1:** Patients’ characteristics

Parameter	Total (%)
Age (years)	
> 60	19 (29.7)
< 60	45 (70.3)
Menopausal status	
Premenopausal	17/64 (26.6)
Perimenopausal	11/64 (17.2)
Postmenopausal	36/64 (56.3)
Histology	
Ductal	44/64 (68.8)
Lobular	1/64 (1.7)
Others	15/64 (23.4)
Unknown	4/64 (6.3)
Tumor grading	
I	0
II	12/64 (18.8)
III	51/64 (79.7)
Unknown	1/64 (1.6)
Ki 67	
0–10%	3/64 (4.7)
11–30%	7/64 (10.9)
> 30%	41/64 (64.1)
Unknown	13/64 (20.3)
Tumor size at diagnosis (c/pT)	
c/pT1a-c	23/64 (35.9)
c/pT2	34/64 (53.1)
c/pT3	4/64 (6.3)
c/pT4	3/64 (4.7)
Tumor size after NACT (ypT)	
ypT0	28/64 (43.7)
ypT1	17/64 (26.6)
ypT2	12/64 (18.7)
ypT3	1/64 (1.6)
ypT4	1/64 (1.6)
Unknown	5/64 (7.8)
Nodal Status at diagnosis (c/pN)	
Node-negative (c/pN−)	44/64 (68.8)
Node-positive (c/pN+)	20/64 (31.3)
Nodal status after NACT (ycN)	
Node-negative (ycN-)	5/64 (7.9)
Node-positive (ycN +)	2/64 (3.1)
na	56/64 (88)
Chemotherapy	
Neoadjuvant	59/64 (93.6)
Adjuvant	4/64 (6.3)
na	1/64 (1.6)
Pathological response	
Complete response	29/64 (45.3)
Partial response	26/64 (40.6)
No response	4/64 (6.4)
na	5/64 (7.8)
Distant metastases	
Yes	9 (14.1)
No	54 (84.4)
Unknown	1 (1.6)
Recurrence (3y-PFS)	
Alive	52 (81.3)
Relapsed	11 (14.1)
Unknown	1 (1.6)
Overall survival (3y OS)	
Alive	56 (87.5)
Dead	7 (10.9)
Unknown	1 (1.6)

*na* not applicable, *c* clinical; *p* pathological; *y* after neoadjuvant chemotherapy

### Eligibility criteria and response criteria

The eligibility criteria were histologically proven early TNBC, no severe uncontrolled comorbidities or medical conditions, no metastasis at the time of diagnosis and no further malignancies at present or in the patients’ history. Furthermore, blood samples were obtained at the time of primary diagnosis and after NACT, if applicable. Patients were treated according to current guidelines (AGO guidelines; https://www.ago-online.de) including NACT and ACT (adjuvant chemotherapy) (anthracyclines, taxanes, cyclophosphamide, carbo- and cisplatin, myocet, gemcitabine) and radiotherapy. Four patients received the PARP-inhibitor Olaparib in a clinical trial (GeparOLA trial); 59 patients received chemotherapy in the neoadjuvant setting; four patients were in the adjuvant setting; one patient received no chemotherapy. The tumor type, TNM-staging, grading and Ki67 were assessed at the Institute of Pathology, at the University Hospital Essen as part of the West German Comprehensive Cancer Centre for each of the 64 patients. Pathological response to chemotherapy was defined according to the grading system of Sinn et al. (1994): 0 = no effect; 1 = resorption and tumor sclerosis, 2 = minimal residual invasive tumor (< 0.5 cm), 3 = residual non-invasive tumor only, ductal carcinoma in situ (DCIS), 4 = no tumor detectable. In our cohort, pCR was defined as regression 4 according to Sinn, no evidence of residual invasive cancer and DCIS, both, in breast and axilla; pathological partial response (pPR) was defined as regression 1–3 according to Sinn. Blood was obtained after written informed consent from all subjects using protocols approved by the clinical ethic committee of the University Hospital Essen (05/2856). Patients’ characteristics before and after NACT are documented in Table 2: About 43.8% of the patients were pre- or perimenopausal. The predominant histological subtype was ductal carcinoma (68.8%). More than 50% of the patients had a T2 tumor and higher at the time of first diagnosis and about 36% presented with a T1 tumor. Nodal status was positive in 31.3% of the cases at the time of diagnosis. Most patients had an aggressive tumor biology with a grade 3 tumor (79.7%) and the majority showed a Ki67 above 30%. Overall, pCR or pPR had been achieved in 45.3% and 40.6% of cases, respectively, whereas 6.4% of the patients did not respond to the given therapeutic regimen.

**Table 2:** Association of Pre-EV, PD-L1_EV_ and PD-L2_EV_ levels with clinical parameters of TNBC patients

Parameter	*n*	pre-PD-L1_EV_ (pg/ml)			pre-PD-L2_EV_ (pg/ml)			*n*^a^	pre-EV level (10^11^/ml)		
		Med	Min	Max	Med	Min	Max		Med	Min	Max
Age (years)											
> 60	15	118	53	243	108	27	321	15	6.60	1.20	15.00
< 60	41	147	43	4245	177	10	2870	40	3.30	1.10	30.00
Menopausal status											
Premenopausal	13	188	65	2188	191	46	2418	13	2.70*^b^	**1.10**	**9.80**
Perimenopausal	10	143	55	334	143	10	1065	10	4.80*^b^	**1.80**	**7.60**
Postmenopausal	33	122	43	4245	135	27	2870	32	6.35*^b^	**1.20**	**30.00**
Histology											
Ductal	37	175	43	4245	158	10	2870	36	4.30	1.10	30.00
Lobular	1	132	132	132	87	87	87	1	2.60	2.60	2.60
Others	15	99	60	275	151	27	547	15	5.80	1.80	9.10
Unknown	3	113	102	140	128	66	132	3	1.90	1.20	2.20
Tumor grading											
I	0							0			
II	12	150	53	3846	130	10	2870	12	2.25	1.20	6.80
III	43	139	43	4245	157	27	2852	42	5.60	1.10	30.00
Unknown	1	60	60	60	91	91	91	1	7.70	7.70	7.70
Ki 67											
0–10%	2	100	60	140	106	84	128	2	4.00	2.20	5.80
11–30%	6	141	103	238	99	10	590	6	6.10	1.40	15.00
> 30%	36	134	43	4245	154	27	2870	35	6.10	1.10	30.00
Unknown	12	179	60	454	191	46	1378	12	3.50	1.80	10.00
Tumor size at diagnosis (c/pT)											
c/p T1a-c	23	141	55	791	143	27	2420	23	4.50	1.30	15.00
c/p T2	28	144	43	4245	149	10	2870	27	5.40	1.10	30.00
c/p T3	3	202	55	239	231	108	321	3	5.80	3.40	14.00
c/p T4	2	81	53	108	115	80	151	2	4.25	1.90	6.60
Tumor size after CT (ypT)											
ypT0	24	108	43	3846	122	10	2870	23	3.40	1.30	30.00
ypT1	14	159	74	2188	136	27	2418	14	4.60	1.10	15.00
ypT2	11	139	53	4245	177	71	2852	11	6.30	1.20	14.00
ypT3	1	183	183	183	191	191	191	1	2.50	2.50	2.50
ypT4	1	249	249	249	210	210	210	1	11.00	11.00	11.00
Unknown	5	201	65	375	440	46	590	5	2.90	1.40	6.60
Nodal status at diagnosis (c/pN)											
Node-negative (c/pN−)	38	120*^c^	**43**	**2188**	134	10	2418	37	4.50	1.10	30.00
Node-positive (c/pN+)	18	197*^c^	**55**	**4245**	183	57	2870	18	4.90	1.30	14.00
Nodal status after CT (ypN)											
Node-negative (ypN−)	3	157	139	243	71	10	89	3	7.60	3.20	8.20
Node-positive (ypN+)	2	2149	53	4245	1466	80	2852	2	7.95	1.90	14.00
na	51	140	43	3846	157	27	2870	51	4.50	1.10	30.00
Chemotherapy											
Neoadjuvant	51	140	43	4245	143	10	2870	50	4.60	1.10	30.00
Adjuvant	4	219	65	375	494	46	590	4	2.15	1.40	5.40
na	1	108	108	108	151	151	151	1	6.60	6.60	6.60
Pathological response											
Complete response	25	113	43	3846	135	10	2870	24	3.95	1.30	30.00
Partial response	22	142	53	4245	138	27	2852	22	4.60	1.10	15.00
No response	4	193	72	223	256	183	1065	4	6.30	2.50	8.30
na	5	201	65	375	440	46	590	5	2.90	1.40	6.60
Distant metastases											
Yes	8	139	65	4245	183	87	2852	8	3.10	1.30	14.0
No	47	141	43	3846	134	10	2870	46	4.60	1.10	30.0
Unknown	1	51	51	51	150	150	150	1	6.60	6.60	6.60

Statistical significance was tested by Mann–Whitney test in case of two groups or Kruskal–Wallis test in cases of more than two groups, levels in bold indicate a significant difference *p < 0.05

*CT* chemotherapy, *Med.* median, *Min* minimum, *Max* maximum, *na* not applicable, *c* clinical; *p* pathological; *y* after neoadjuvant chemotherapy

^a^One preparation of EV was not available for NTA measurement

^b^Significance obtained according Kruskal–Wallis test

^c^Significance obtained according Mann–Whitney test

### Ethics approval and consent to participate

The study was conducted according to the guidelines of the Declaration of Helsinki, and approved by the Institutional Review Board of University Hospital Essen (05/2856). Informed consent was obtained from all subjects involved in the study.

### Sampling of blood

Ethylene-diamine-tetra-acetic acid (EDTA) blood was collected for the isolation of CTC before the application of therapeutic substances with an S-Monovette (Sarstedt AG & Co.) and stored at 4 °C until further examination. The samples were processed within 4 h after blood collection. EDTA samples from 16 age-matched healthy females [median (range): 47 (35–62) years] served as control panel. Plasma samples of each patient were generated from EDTA blood and centrifuged at 1500 × *g* for 10 min. Subsequently, the upper phase was stored at − 80 °C until usage.

### Isolation and characterization of extracellular vesicles

As recently described, EV were enriched from plasma samples by ExoQuick™ (SBI Systems Bioscience Inc., Mountain View, VA, USA). Before EV precipitation by ExoQuick™, all samples were spun down at 3000 g for 15 min. Subsequently, 250 μl of plasma supernatant was added to 63 μl ExoQuick™ reagent and incubated over night at 4 °C. Thereafter, the samples were centrifuged at 1500 g for 30 min in the cold (4 °C), supernatants were discarded from the samples and again centrifuged at 1500 g for 5 min in the cold. The remaining pellets containing the EV were re-suspended in 250 μl 0.9% NaCl and stored at -20 °C. Characterization of EV preparations was performed by nano-tracking analysis using ZetaView Laser Scattering Video Microscope (Particle Metrix GmbH, Microtrac, Meerbusch, Germany) and its corresponding software (version 8.03.08.02). To this end, EV preparations were regularly diluted 1:50,000 in PBS to obtain particle concentrations of approx. 1 × 10^6^ per ml (Sokolova et al. 2011; Konig et al. 2016, 2017; Schwich et al. 2019). The coefficients of variation among different Zeta-View measurements were 24.8% for the enumeration and 6.7% for the size.

### Quantitation of vesicular PD-L1 and PD-L2

Amounts of PD-L1 and PD-L2 bound to EV (PD-L1_EV_ and PD-L2_EV_) were quantified in the EV-enriched preparations undiluted by commercial ELISA kits (R&D Systems, GmbH, Wiesbaden-Nordenstadt, Germany), as previously described (Buderath et al. 2019; Ugurel et al. 2020). Microtiter plates having high binding surface (Costar Corning, Bodenheim, Germany) were coated with anti-human PD-L1 or PD-L2 antibody at 4 °C overnight in a final concentration of 4 µg/ml and 2 µg/ml, respectively. A biotin-coupled polyclonal goat anti-human PD-L1 or PD-L2 antibody diluted in phosphate-buffered saline (PBS) served as detection reagent, supplemented with 1% bovine serum albumin (BSA, AppliChem GmbH, Darmstadt, Germany) to a final concentration of 50 ng/ml and 1 µg/ml, respectively. Bound antibody was recognized by streptavidin conjugated with horseradish peroxidase being diluted 1:200 in PBS containing 1% BSA. 3,3,5,5-tetramethybenzidine substrate reagent set (Becton Dickinson, Franklin Lakes, USA) was used for visualizing immune complexes. Substrate reaction was stopped using 2 N H2SO4, and optical density was measured at 450 nm (Biotek Instruments, Winooski, VT). EV preparations were tested undiluted. Recombinant PD-L1 and PD-L2 protein fused with Fc portion of human IgG were used as standard reagents. PD-L1 and PD-L2 standard reagents were serially diluted from 0 to 1,250 pg/ml or 0 to 6,000 pg/ml. Quantifications of PD-L1_EV_ and PD-L2_EV_ levels were performed by four-parameter curve fitting. For PD-L1 and PD-L2 ELISA formats, the intra-assay coefficients of variations were 6.6 and 5.2%, respectively, whereas the inter-assay coefficients of variations were 15.0% for PD-L1 and 9.1% for sPD-L2.

### Selection and detection of CTC

CTCs were isolated from 2 × 5 ml EDTA blood by positive immuno-magnetic selection targeting EpCAM, EGFR and HER2 (AdnaTest EMT-2/StemCell SelectTM, QIAGEN GmbH, Hilden, Germany) as recently described (Bittner et al. 2020; Kasimir-Bauer et al. 2020). Briefly, labeled CTCs were extracted using a magnetic particle concentrator and were lysed according to the manufacturer`s instructions. mRNA was isolated from the resulting cell lysates by oligo(dT)25-coated magnetic beads and reverse-transcribed (AdnaTest EMT-2/StemCell DetectTM, QIAGEN GmbH, Hilden, Germany) with a final reaction volume of 40 µl. cDNA was stored at − 20 °C.

To detect different CTC subpopulations, including AKT2, ALK, AR, AURKA, BRCA1, EGFR, ERCC1, ERBB2, ERBB3, KIT, KRT5, MET, MTOR, NOTCH1, PARP1, PIK3CA, SRC and GAPDH, multi-marker RT-qPCR panels (QIAGEN GmbH, Hilden, Germany) were used for 46 TNBC patients pre- and for 44 post-CT. The methods require transcript-specific pre-amplification of 6.25 µl cDNA using Multiplex PCR Master Mixes (QIAGEN GmbH, Hilden, Germany) with 18 PCR cycles. Pre-amplified cDNA (2 µl; 1:10 diluted) was used in duplicates for one of the 18 transcripts in a reaction volume with SYBR Green-based components in total of 10 µl. RT-qPCR was performed with the StepOnePlus™ (Thermo Fisher Scientific, Waltham, USA) real-time system. CTC expression data were normalized to matched expression data of healthy donor controls. CTC isolation was performed in duplicate for each patient and cDNA was analyzed separately from these duplicates. After binary evaluation of the qPCR data, signals per patient were regarded positive if at least one of the sample duplicates showed a positive ∆(∆)Cq value. Establishment of the method including data evaluation has been described in detail recently (Kasimir-Bauer et al. 2020). Furthermore, application of this method in blood samples of the current patients’ population including all raw data showing the Cq values of all patients’ samples and healthy donors is elucidated in Bittner et al. 2020.

### Statistical analysis

All statistical analyses were performed using IBM SPSS Statistics Version 23 and GraphPad Prism V8.43 software (GraphPad Software, San Diego, CA, USA). With the exception of the size of EV, which is presented as mean ± SD, all metric parameters are given as median and range. After testing for distribution, continuous and categorical variables were compared using Mann–Whitney *U*, Kruskal–Wallis test, Fisher’s exact test or Chi-square test, as appropriate. Receiver operating curve (ROC) analysis was performed to calculate optimal cut-off values concerning sensitivity and specificity for stratifying continuous parameters into dichotomous variables, using the BIAS 11.10 software program (http://www.bias-online.de/). Probabilities of OS and PFS were analyzed using the Kaplan–Meier method in combination with the Mantel–Cox log-rank test implemented in the R package survminer (version 0.4.0; https://CRAN.R-project.or/package=survminer). For patients, groups > 2 multiple comparison of OS and PFS probabilities among groups were performed using the Peto-Pike log-rank test (BIAS 11.10 software program: http://www.bias-online.de). Starting points were time point of diagnosis (blood collection) and endpoints were death from BC and relapse of BC. Differences with a p value < 0.05 were considered statistically significant.

## Results

### Increased extracellular vesicles in plasma of TNBC

**Fig. 1:**
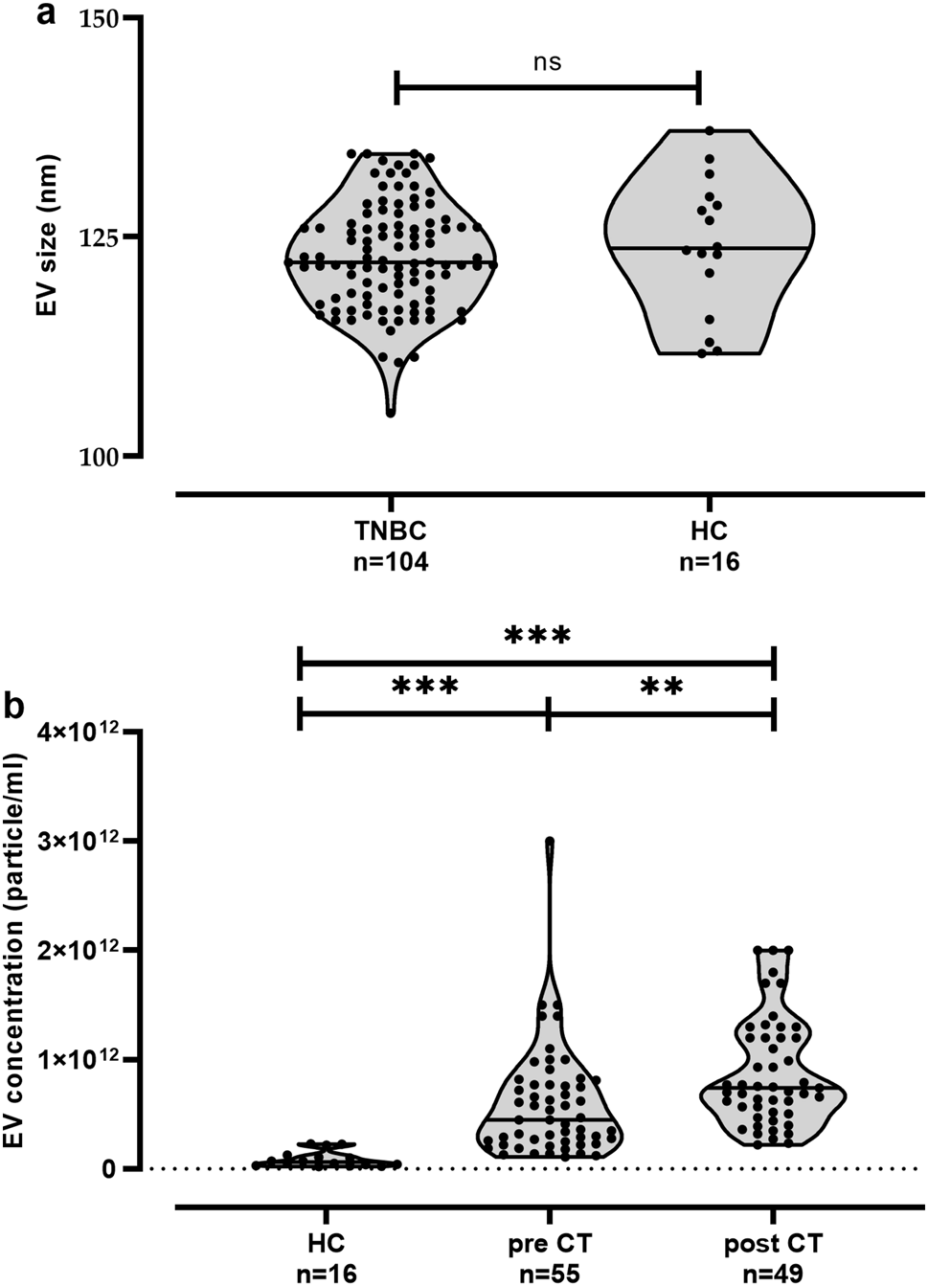
Size and particle numbers of plasma-derived extracellular vesicles in HC and TNBC patients. **a** Particle size distribution in TNBC patients and healthy controls (HC) and **b** EV plasma concentration in HC and in patients’ pre- and post-chemotherapy (CT). EV size and levels were defined by NTA. EV preparations were not available for one pre- and for one post-CT patient for NTA measurement. Straight lines within violins indicate the median. Statistical significance was tested by Mann–Whitney test (**a**) or Kruskal–Wallis test (**b**); *ns* not significant; ***p < 0.001; **p < 0.01

The size and the EV particle concentration of the ExoQuick™ preparations were determined from TNBC patients and female healthy controls (HC) by Nanotracking Analysis (NTA). The results revealed a nearly identical size distribution (mean ± SD nm) for EV preparations derived from plasma samples of TNBC patients (122. ± 5.99) and HC (123.9 ± 7.79, Fig. 1a), both being in range (50–150 nm) of the acknowledged size of exosomes (Gyorgy et al. 2011). However, levels of EV [median (range) 1011/mL] obtained from plasma samples of TNBC patients pre [4.5 (1.1–30.0), *n* = 55]- and post-CT [7.4 (2.2–20.0), *n* = 49] were more than tenfold (*p* < 0.0001) increased compared to those of HC [0.6 (0.2–2.3), *n* = 30, Fig. 1b]. Moreover, post-CT EV levels were significantly (*p* = 0.002) higher than the one obtained from patients pre-CT. In addition, pre-EV levels were significantly increased in patients with postmenopausal status compared to patients having a pre- or perimenopausal status (*p* = 0.017, Table 2). No further association to clinical parameters could be identified (Table 2), even for post-EV concentration (data not shown).

### Association of increased pre-CT PD-L1_EV_ levels with positive nodal status of TNBC patients at time of diagnosis

**Fig. 2:**
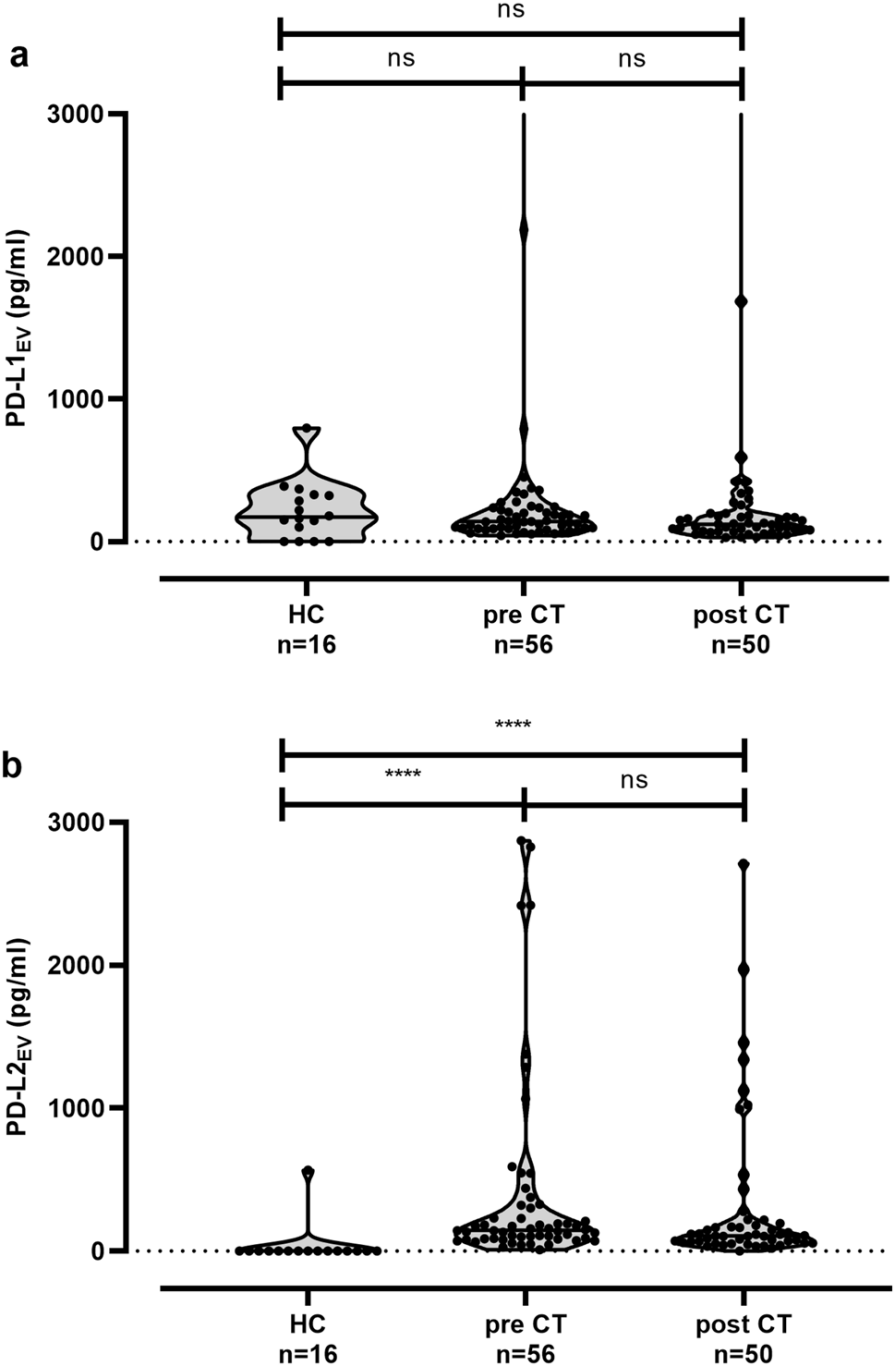
Comparison of PD-L1_EV_, PD-L2_EV_ in plasma samples of HC and TNBC patients. **a** Levels of PD-L1_EV_ were not significantly different in patients’ pre- and post-chemotherapy (CT) and healthy controls (HC), whereas (**b**) levels of PD-L2_EV_ were significantly increased in pre- and post-CT patients compared to HC. Straight lines within violins indicate median values. Statistical significance was tested by Kruskal–Wallis test; ns: not significant; ****p < 0.0001

We further determined levels of EV harboring the PD-L1 (median [range] pg/ml). Levels of pre [141 (43–4285), *n* = 56]- and post-CT PD-L1_EV_ [122 (73–3903), *n* = 50] were similar to the ones of HC [172 (0–797), *n* = 16; Fig. 2a]. Moreover, PD-L1_EV_ of pre (*r* = 0.055, *p* = 0.689)- and post-CT PD-L1_EV_ levels (*r* = − 0.210, *p* = 0.169) did not correlate with EV particle concentration in the corresponding EV preparations. However, pre-PD-L1_EV_ levels were significantly elevated (*p* = 0.022) in patients with a positive nodal status at first diagnosis [197 (55–4245), *n* = 18] as compared to patients with a negative nodal one [120 (43–2188), *n* = 38, Table 2]. No further relationship between post PD-L1_EV_ and clinical parameters could be established (data not shown).

### Increased levels of PD-L2_EV_ in TNBC patients

In contrast to PD-L1_EV_, PD-L2_EV_ levels were significantly (*p* < 0.0001) increased in TNBC patients compared to HC (0 [0–567]). However, pre- and post-CT PD-L2_EV_ levels did not substantially vary (Fig. 2b). Additionally, pre (*r* = − 0.012, *p* = 0.929)- and post-CT PD-L2_EV_ levels (*r* = − 0.136, *p* = 0.342) did not correlate with levels of EV particles. Of note, PD-L2_EV_ could only be detected in one out of 16 EV preparations derived from plasma samples of HC, whereas with one exception, all EV preparations derived from pre- and post-CT plasma samples did contain PD-L2_EV_ (*p* < 0.0001).

### Association of reduced PD-L2_EV_ levels post CT with complete response

**Fig. 3:**
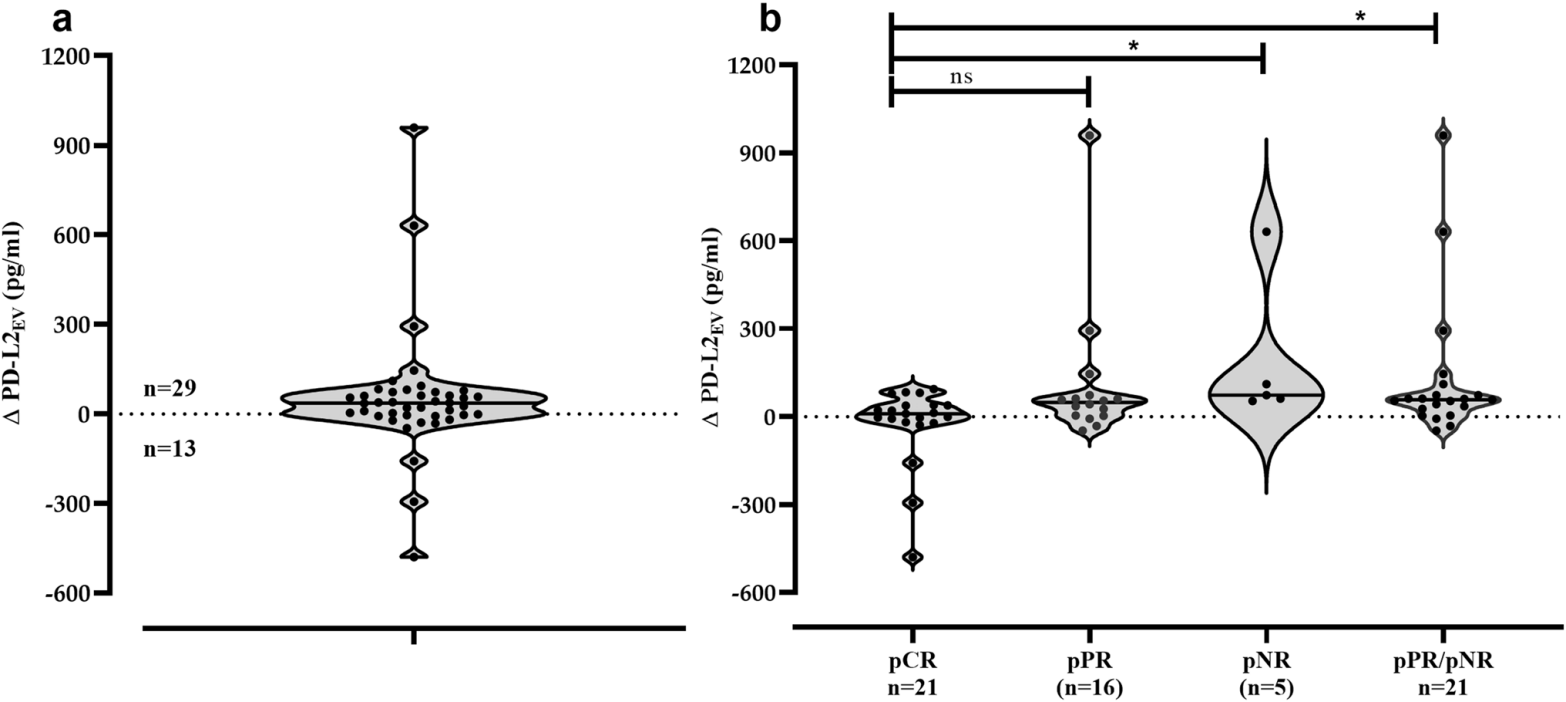
Difference of pre- and post-PD-L2_EV_ levels and its association with CT outcome. **a** PD-L2_EV_ differences (Δ PD-L2_EV_) were calculated between pre- and post-chemotherapy (CT) levels in plasma samples of paired TNBC patients (*n* = 42). Obviously, 13 patients revealed a loss of PD-L2_EV_ and 29 patients showed an increase of PD-L2_EV_ post CT. **b** The Δ PD-L2_EV_ levels were stratified according to the patients’ CT response. Using Kruskal–Wallis test with Dunn’s test for multiple comparison, ∆PD-L2_EV_ levels of patients with complete response **(**pCR) post CT were significantly lower than the ones of patients who did not response to CT, whereas the ∆PD-L2_EV_ levels did not differ between patients with pCR and patients with pathological partial response (pPR). Overall, ∆PD-L2_EV_ levels were significantly reduced in patients with pCR compared to patients with pPR/pNR by Mann–Whitney test. Straight lines within violins indicate median values. *p < 0.05

To analyze the effect of CT on PD-L2_EV_ levels, the differences between PD-L2_EV_ levels of pre- and post-CT PD-L2_EV_ levels (Δ PD-L2_EV_) were defined in paired plasma samples of TNBC patients (*n* = 42). A clear reduction of PD-L2_EV_ levels (-Δ PD-L2_EV_ values) post CT was observed for 13 out of the 42 TNBC patients (Fig. 3a).

Stratification of patients according to their therapy response (Fig. 3b) evidenced that a loss of PD-L2_EV_ was significantly (*p* = 0.048, Chi-square test) more prominent in patients with pCR (10 out of 21, 47.6%) compared to patients with pPR (3 out of 16, 18.8%) and compared to patients with pathological non-response (pNR; 0 out 5, 0%). Regarding $\Delta$ PD-L2_EV_ levels [median (range) pg/ml], patients with pCR had a significant (*p* = 0.013, Kruskal–Wallis test) lower level [10 (− 480 to 574), *n* = 21] than patients with pPR [48 (−48 to 956), *n* = 16] or patients with pNR [73 (53–577), *n* = 5]. The multiple comparison by Dunns test revealed that the median of ∆PD-L2_EV_ obtained from patients with pCR was significantly reduced compared to median of patients with pNR (*p* = 0.011) but not reduced compared to median of patients with pPR (*p* = 0.171). At contrast to PD-L2_EV_, a reduction of EV particle concentration or PD-L1_EV_ levels post CT was less frequent and not associated with pCR (data not shown).

### Association of increased pre- and post-CT EV, PD-L1_EV_ or PD-L2_EV_ levels with distinct CTC

**Fig. 4:**
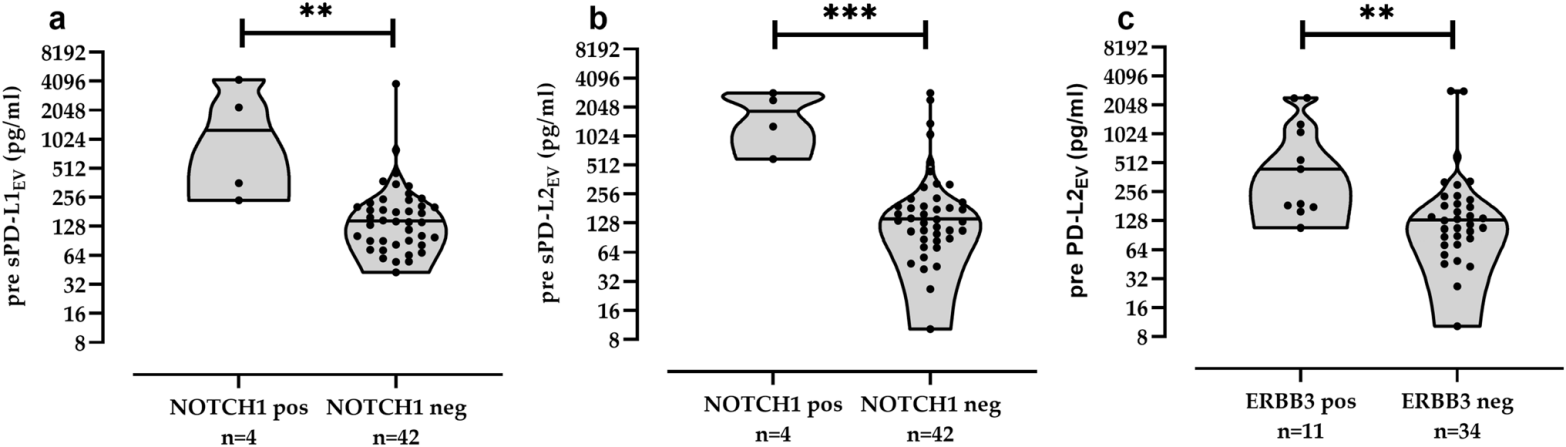
Association between CTC subpopulations and levels of PD-L1_EV_ and PD-L2_EV_ pre-CT. Increased PD-L1_EV_ (**a**) and PD-L2_EV_ levels (**b**) were found in patients with NOTCH1-positive CTC, and elevated PD-L2_EV_ levels (**c**) were observed for patients displaying ERBB3-positive CTC compared to patients being negative for these CTC subpopulations. ERBB3 CTC data were not available for one pre-CT patient. Data of PD-L1_EV_ and PD-L2_EV_ are presented in log2 scale due to the high variability of levels. Straight lines within violins indicate median values. Statistical significance was tested by Mann–Whitney test, **p < 0.01, ***p < 0.001

As EV and subpopulations of PD-L1_EV_/PD-L2_EV_ provide tumor-supporting characteristics within the tumor microenvironment [2–5], their pre- and post-CT levels were analyzed with respect to presence or absence of specific CTC subpopulations including AKT2, ALK, AR, AURKA, BRCA1, KIT, MET, EGFR, ERCC1, ERBB2, ERBB3, KRT4, mTOR, NOTCH 1, PARP1, PIK3CA, SRC. CTC subpopulations pre-CT were not associated with increased EV particle levels (data not shown). However, pre-CT median levels of PD-L1_EV_ [1274 (238–4245), *n* = 4] and PD-L2_EV_ [1852 (589–2872), *n* = 4] were more than eightfold elevated in patients with NOTCH1-positive CTC (*n* = 4) compared to the ones (*n* = 42) with CTC not expressing NOTCH1 (Fig. 4a,b). Moreover, pre-CT PD-L2_EV_ levels were significantly increased (*p* = 0.003) in patients with ERBB3-positive CTC [440 (108–2420), *n* = 11] compared to patients without ERBB3-positive CTC [131 (10–2870), *n* = 34; Fig. 4c].

**Fig. 5:**
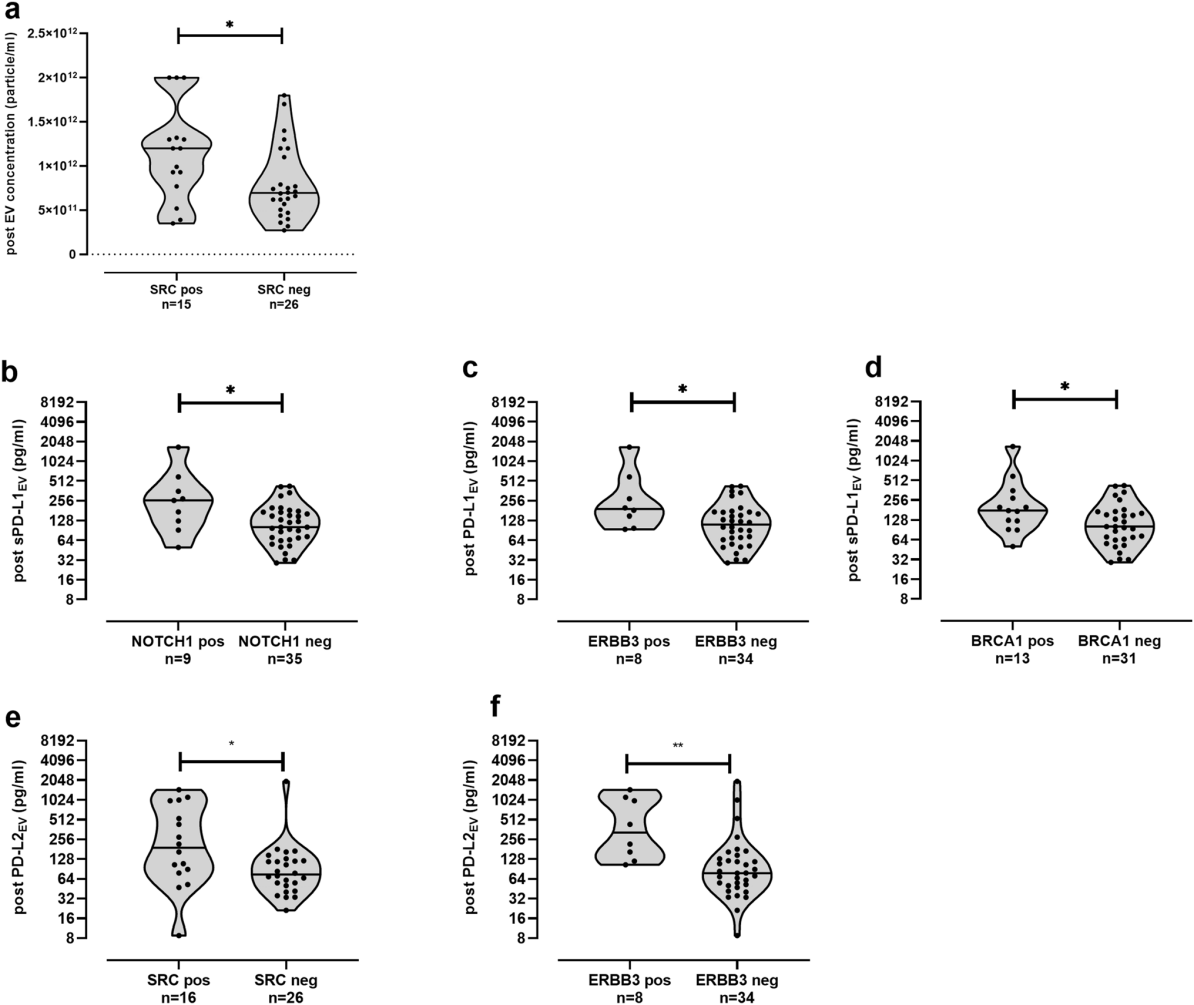
Association between CTC subpopulations and EV particles, PD-L1_EV_, and PD-L2_EV_ levels post chemotherapy. Increased EV particle concentration (**a**), PD-L1_EV_ (**b**, **c**) or PD-L2_EV_ levels (**e**–**f**) were found in patients with SRC-positive (**a**, **e**) or NOTCH1-positive (**b**), ERBB3-positive (**c**, **f**), BRCA1-positive (**d**) CTC compared to corresponding negative CTC patients post chemotherapy. Not all CTC data (SRC, ERBB3) were available post CT. Data of PD-L1_EV_ and PD-L2_EV_ are presented in log2 scale due to the high variability of levels. Straight lines within violins indicate median values. Statistical significance was tested by Mann–Whitney test, *p < 0.05, **p < 0.01

For the post-CT situation (Fig. 5), an association of increased EV particle levels (*p* = 0.03) was observed in patients with SRC-positive CTC [12.0 (3.5–20), *n* = 15] compared to patients without SRC-positive CTC [7.0 (3.0.0–20.0), *n* = 26; Fig. 5a]. Comparable to the pre-CT situation, post-PD-L1_EV_ was elevated in patients with NOTCH1-positive CTC [259 (50–1685), *n* = 9; *p* = 0.03)], and PD-L2_EV_ levels were higher in patients harboring ERBB3 [356 (105–1458), *n* = 8, *p* = 0.0014]-positive CTC compared to corresponding NOTCH1-negative [102 (29–427), *n* = 35; Fig. 5b] or ERBB3-negative CTC [79 (9–1970), *n* = 34, Fig. 5f] patients. In addition, PD-L1_EV_ levels were significantly (*p* = 0.03) higher in patients with ERBB3- [191 (94–1685), *n* = 8] or with BRCA1-positive CTC [177 (51–1685), *n* = 13] than in patients with CTC not expressing ERBB3 [111 (29–427), *n* = 34] or BRCA1-[102 (29–427), *n* = 31] (Fig. 5c,d). Similar to EV particle concentration (Fig. 5a), significant increased levels of PD-L2_EV_ [191 (9–1458), *n* = 16] could be identified for SRC CTC-positive patients versus to the SRC CTC-negative patients subpopulation [75 (21–1970), *n* = 26, Fig. 5e].

### Association of high pre-CT PD-L2_EV_ levels with inferior PFS and OS in TNBC patients

**Fig. 6:**
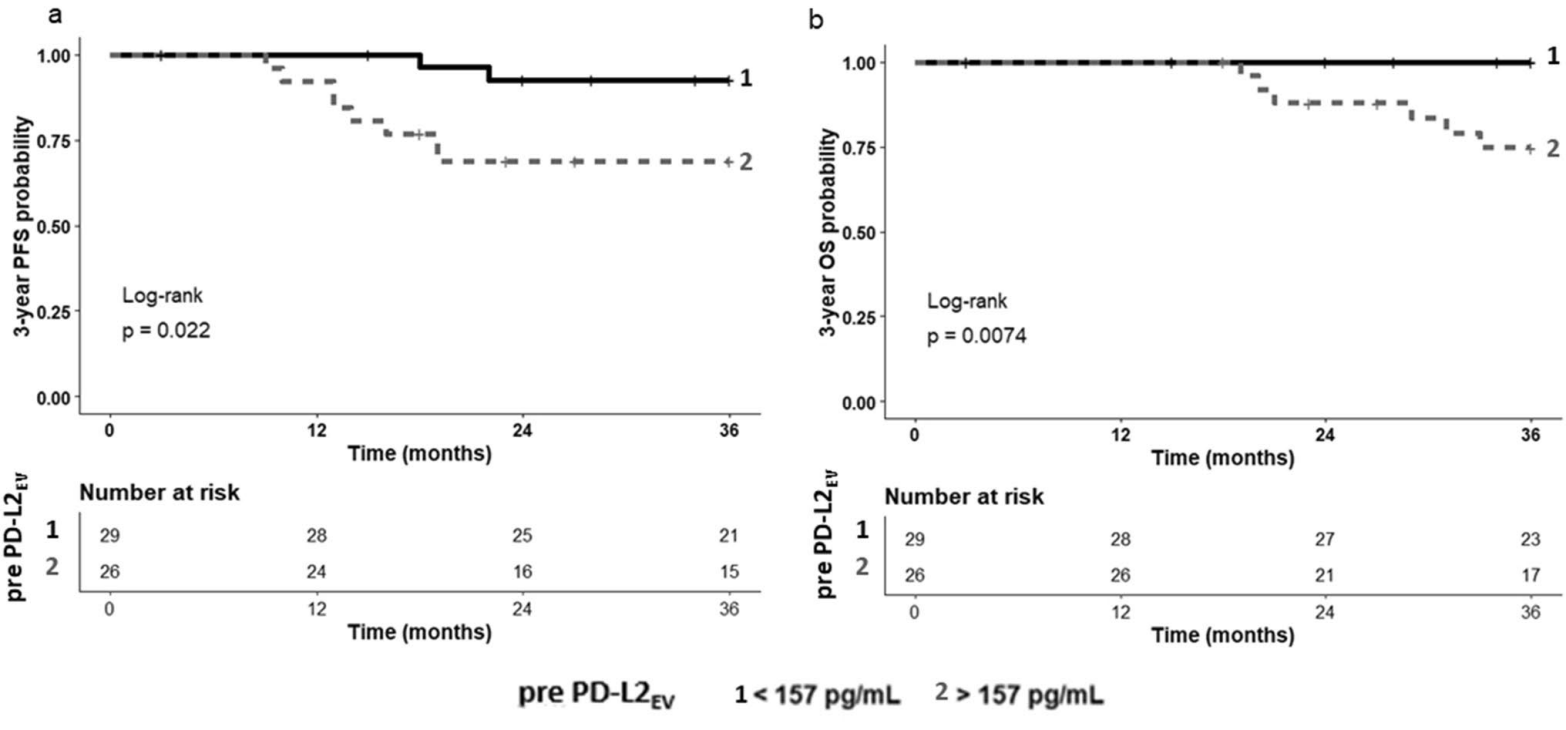
Association of high PD-L2_EV_ levels pre-CT with reduced 3-year PFS and OS. Patients were divided in two groups according to cut-off PD-L2_EV_ levels (< 157 pg/ml). The corresponding Kaplan–Meier curve of 3-year PFS and OS probability combined with Mantel–Cox log-rank test demonstrated a reduced probability of PFS (**a**, *p* = 0.022) or OS (**b**; *p* = 0.0074) for patients with PD-L2_EV_ levels above the threshold (2 in gray dashed) compared to patients with PD-L2_EV_ levels below this value (1 in black solid) pre-CT. Time was calculated from blood sampling to event (progression/death)

For pre- and post-EV particle concentration and PD-L1/L2_EV_ levels, ROC analyses were performed to define the best threshold value regarding the prediction of PFS and OS of TNBC patients after CT. A pre-CT PD-L2_EV_ cut-off level of 157 pg/ml was related to the probability of PFS (AUC: 0.698; sensitivity: 80%, specificity: 60%; *p* = 0.021) and OS (AUC: 0.738; sensitivity: 100%, specificity: 59.2%; *p* = 0.008), whereas no relevant cut-offs could be identified for the other pre- or post-CT markers (data not shown). Using the pre-CT PD-L2_EV_ threshold, the Kaplan–Meier 3-year probabilities of PFS (Fig. 6a) and of OS (Fig. 6b) were significantly reduced for patients above threshold compared to patients below this level (*p* = 0.022; Mantel–Cox log-rank HR: 5.05, 95% CI: 1.45–17.60 and *p* = 0.0074; Mantel–Cox log-rank Hazard Ratio (HR): 8.98, 95% confidence interval (CI): 1.80–44.83, respectively).

### Identification of TNBC patients with early risk of progression by the presence of high PD-L2_EV_ levels and ERBB3-positive CTC pre-CT

**Fig. 7:**
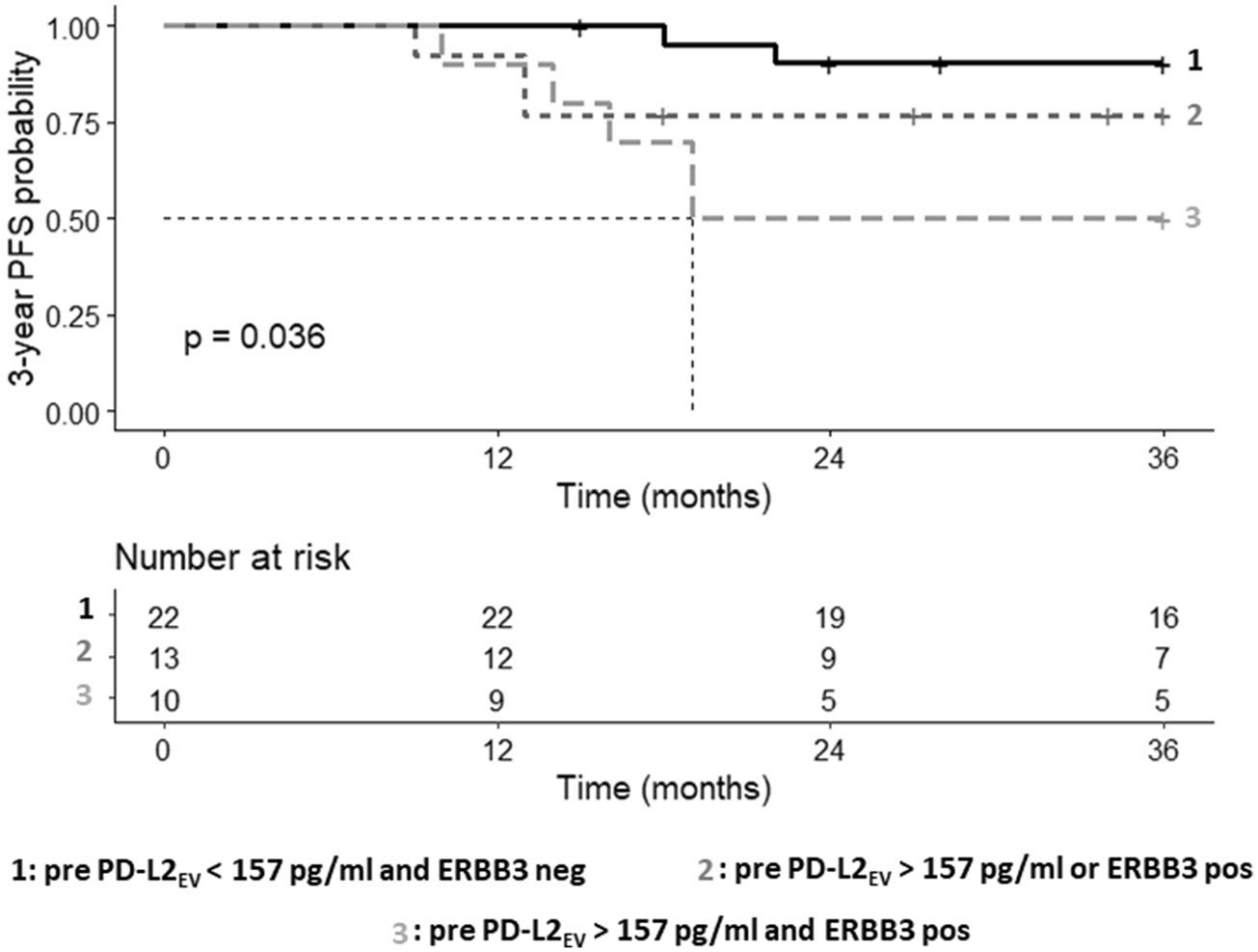
High CT PDL-2_EV_ combined with ERBB3-positive CTC identifies TNBC patients with high risk of early progression. Patients were divided into three groups: Group 1 (in gray solid), comprising patients with PD-L2_EV_ > 157 pg/ml and with ERBB3-positive CTC pre-CT; group 2 (in dark gray dashed), comprising patients with either PD-L2_EV_ > 157 pg/ml or with ERBB3-positive CTC; group 3 (in black dashed), comprising patients with PD-L2_EV_ levels < 157 pg/ml and absence of ERBB3-positive CTC. Kaplan–Meier curve of 3-year progression-free survival (PFS) probability combined with Mantel–Cox log-rank test revealed significantly reduced PFS probabilities in these groups. Dotted line indicates the median survival time. Time was calculated from blood sampling to event (progression). Overall p values are shown

As high PD-L2_EV_ were associated with the presence of ERBB3-positive CTC, patients were stratified by having PD-L2_EV_ < 157 pg/ml and no ERBB3-positive CTC (group 1), having either PDL-2_EV_ > 157 pg/ml or ERBB3-positive CTC (group 2), and having PD-L2_EV_ > 157 pg/ml and ERBB3-positive CTC (group 3) pre-CT. These patients’ groups presented different PFS probabilities (p = 0.036, log-rank [Mantel-Cox] test, Fig. 7). According to multiple comparison by Peto-Pike log-rank test, the patients’ group 3 (PD-L2_EV_ > 157 pg/ml and ERBB3-positive CTC) displayed with a median PFS time of 19 months a shortened PFS probability compared to the patients’ group 1 (HZ: 6.97, 95% CI: 1.71- 28.50, *p* = 0.007, *p*corr = 0.02). However, these two groups were not significantly different concerning OS probabilities (data not shown). Even more, the stratification according to PD-L2_EV_ and ERBB3 status post CT as well as the stratification of PD-L2_EV_ and NOTCH1 CTC status pre/post-CT did not identify patients with high risk of early progression or a reduced OS (data not shown).

## Discussion

In early TNBC, the choice of immunotherapy in combination with chemotherapy is a promising therapeutic option to significantly improve pCR and EFS (Mittendorf et al. 2020; Schmid et al. 2020a, b, c) and probably OS.

Thus, minimally invasive biomarkers are urgently needed in TNBC, not only for identification of patients with high risk for recurrence, but also as a selection criterion of immune therapeutic approaches or of patients benefiting from PD-1 ICI or other targeted immunotherapies. Based on the tumor-supporting roles of EV or of certain EV subpopulations, we hypothesized that EV particle concentrations or levels of PD-L1/2-bearing EV in liquid biopsies of plasma samples may function as useful surrogate markers in TNBC for disease outcome, alone or in combination with distinct CTC subpopulations.

Interestingly, the most important clinical implications could be demonstrated for PD-L2_EV_ but rarely for PD-L1_EV_ levels, which were only elevated in node-positive patients before the onset of therapy. In contrast, as compared to HC, TNBC patients had significantly elevated PD-L2_EV_ levels as well as a tenfold higher EV concentration. Pre-CT, PD-L2_EV_ levels > 157 pg/ml were associated with a significantly reduced 3-year probability of PFS and OS compared to patients below this level and a loss of PD-L2_EV_ after CT was significantly more prominent in patients achieving a pCR. With regard to CTC subtypes, the presence of ERBB3-positive CTC in combination with pre-CT PD-L2_EV_ > 157 pg/ml resulted in a shorter PFS probability as compared to patients not harboring these characteristics. Although patients harboring NOTCH1-positive CTC had eightfold elevated levels of PD-L2_EV_, a stratification according to the PD-L2_EV_ and NOTCH1 status pre-CT was not meaningful for the identification of patients with high risk of early progression or a reduced OS (data not shown).

Higher EV concentrations in our patients’ cohort are in line with our recently published data for advanced, non-metastatic BC patients (Konig et al. 2017) and has been confirmed in other BC studies (Galindo-Hernandez et al. 2013) as well as in pancreatic (Melo et al. 2015) and lung cancer studies (Choi et al. 2020). For tumor-derived EV, hypoxia or stress in microenvironment of the tumors are crucial factors leading to an increased release of EV. Furthermore, EV production and secretion appeared to be under the control of p53 protein, Heparanase, or Rap GTPase proteins (Whiteside 2016; Szabo and Momen-Heravi 2017).

The fact that only PD-L2_EV_ levels, not PD-L1_EV_ levels were significantly increased is of certain interest, since most of the published data refer to the relevance of PD-L1 concerning response to therapy and clinical outcome (Sadovska et al. 2015; Chen et al. 2018; Ugurel et al. 2020). In head and neck cancer, the level of PD-L1 containing exosomes significantly correlated with clinico-pathological parameters, such as disease activity, UICC stage and lymph node status (Theodoraki et al. 2018). In our patients, except for significantly elevated pre-PD-L1_EV_ levels in node-positive patients, no further association of PD-L1_EV_ levels and clinico-pathological parameters were found. We could also not demonstrate an association of PD-L1_EV_ with response to CT. Our data of vesicular PD-L1 are in line with recently published clinical trials in early TNBC receiving NACT in combination with immunotherapy. These studies clearly demonstrated that a clinical benefit was independent of PD-L1 expression on tumor cells. At variance to early TNBC, a clinical benefit was observed for metastatic TNBC patients expressing PD-L1 on tumor cells (Cortes et al. 2020; Mittendorf et al. 2020; Schmid et al. 2020a, b, c; Schmid et al. 2021).

Remarkably, the presence of PD-L2_EV_ was nearly exclusively observed in TNBC patients and not in HC. For our patients, a loss of PD-L2_EV_ after CT was significantly more prominent in patients achieving a pCR as compared to patients with residual tumor load (pPR or nPR). Comparable data were shown in head and neck squamous cell carcinoma with both, PD-L1 and PD-L2 positivity in tumor, stromal and immune cells significantly predicting clinical response to pembrolizumab (Yearley et al. 2017). Probably, PD-L2_EV_ could function as a marker for therapy response in TNBC. Despite this correlation, we further showed a prognostic association of enhanced PD-L2_EV_ levels prior to therapy and a reduced 3-year probability of PFS and OS. Comparable results were shown for the PD-L2 expression in solid tumor tissue by a meta-analysis including 3533 patients (Yang et al. 2019). This study demonstrated that PD-L2 overexpression was a weak negative predictor for OS and a strong predictor for poor PFS. Among the different tumor entities, patients suffering from hepatocellular carcinoma and clear cell renal cell carcinoma had the most unfavorable outcome presenting with high PD-L2 expression in tumor tissues. Moreover, PD-L2 expression appears to be associated with the presence of lymphatic metastasis. At contrast to these studies, a recent study demonstrated an enhanced mRNA expression of PD-1, PD-L1, and PD-L2 in breast cancer patients post CT or post hormonal therapy, which could be associated with a more beneficial OS even in multivariate analysis (Karsono et al. 2021). A further study analyzing prognostic influence of residual tumor-infiltrating lymphocyte subtypes and the PD-1, PD-L1, and PD-L2 expression in TNBC post CT evidenced that most of the patients presented low PD-L2 expression compared to PD-L1. However, neither PD-1 nor PD-L1/2 expressions were found to be of prognostic relevance for EFS or OS (da Silva et al. 2021). Here, we have to point out that the results on expression in tumor tissues cannot be compared one-to-one with our results, as the sources of circulating EV and EV subpopulations in the blood are extremely divers, e.g., tumor tissue, healthy tissue cells, stroma cells as well as immune cells.

Several studies focus on CTC and EV, as well as their surface proteins, bound or soluble in liquid biopsy to identify patients being eligible for additional treatment options. Besides the high prognostic value of CTC counts in primary and metastatic cancer, the characterization of CTC has become important to identify targets with drugs available to eliminate these cells (Schochter et al. 2019; Menyailo et al. 2020; Chantzara et al. 2021). In the context of PD-L1/2 expression on CTC, only two studies have already addressed this topic in BC. PD-L1 expression on CTC has been demonstrated in a small group of 16 HR-positive, HER2-negative metastatic BC patients at different expression levels in 11/16 cases (Mazel et al. 2015). In a more comprehensive BC analysis, including 100 early and 98 metastatic BC patients, a low concordance between PD-L1 and CD47 on CTC and tumor tissue as well as on peripheral blood mononuclear cells and tumor-infiltrating lymphocytes was observed. In metastatic cases, the presence of CTC with high CD47 or PD-L1 expression was associated with disease progression, shorter PFS and independently predicted an increased risk for relapse and death (Papadaki et al. 2020). Although not including PD-L1/2 in our recently published comprehensive CTC analysis for the presented cohort of TNBC patients, EGFR + / ERBB2 + /ERBB3 + CTC pre-CT and ERBB2 + /ERBB3 + CTC post-CT significantly correlated with a shorter PFS (Bittner et al. 2020). In the current study, especially the presence of ERBB3-positive CTC in combination with PD-L2_EV_ levels above157 pg/ml significantly indicated a shortened PFS probability compared to patients not harboring these characteristics. The ERBB family has been linked to resistance in BC and treatment failure and might explain the worse outcome of this subgroup of patients (Liu et al. 2007; Amin et al. 2010). Furthermore, our trial revealed a more than eightfold elevated median levels of PD-L1_EV_ and PD-L2_EV_ in NOTCH1-positive CTC pre-CT reflecting another mechanism of resistance, associated with a poor OS when overexpressed (Zhang et al. 2019). In metastatic BC, a variety of studies have evaluated therapies targeting NOTCH signaling. In this context, γ‑secretase inhibitors displayed synergistic activity with docetaxel and showed significant antitumor activity, especially in a patient with TNBC (Locatelli et al. 2017). In addition, NOTCH seems to play a key role in BC immunotherapy where NOTCH depletion resulted in an improved efficacy of nivolumab (anti‑PD‑1 antibody) and ipilimumab (cytotoxic T cell‑associated antigen‑4 (CTLA‑4) antibody) (Qiu et al. 2018)).

Regarding PD-L1 expression on EV, cell culture experiments demonstrated that BC-derived PD-L1-bearing EV can bind to PD-1, thus, inhibiting T cell activation and suppressing T cell killing of BC cells. In addition, these subpopulation of EV was able to transport PD-L1 from PD-L1-positive to PD-L1-negative BC cells, highlighting their role in immune evasion of tumor cells (Yang et al. 2018). In this context, it is of note that PD-L2 displays up to six-fold higher affinity to PD-1 than PD-L1 (Keir et al. 2008).

We are aware that our study has several limitations. First, the results based on a mono-centric patients’ cohort of 56 patients have to be validated in larger studies as well as in multi-center studies. Secondly, we did not compare our results to the expression of PD-L1 and PD-L2 on tumor tissue of the patients, this could be enrolled in further trials. Finally, the functional role of sPD-L2 remains to be further elucidated.

## Conclusion

Nevertheless, we here introduced PD-L2_EV_ as a new biomarker to identify early TNBC patients at high risk for relapse. In contrast to PD-L1_EV,_ which was only elevated in node-positive patients’ pre-CT, PD-L2_EV_ levels were associated with response to therapy, PFS, OS and linked to CTC that harbored resistant character. Thus, this study highlights PD-L2_EV_ as a promising biomarker for risk assessment of TNBC patients and represents the basic for additional studies introducing PD-L2_EV_ as an eligibility criterion for PD-1 ICI approaches.

## Supplementary Information

Below is the link to the electronic supplementary material.

Supplementary file1 (TIF 198 KB)

## Abbreviations

ACT: Adjuvant chemotherapy
BC: Breast cancer
CT: Chemotherapy
CTC: Circulating tumor cells
DCIS: Ductal carcinoma in situ
EFS: Event free survival
EV: Extracellular vesicles
HC: Healthy controls
Her2: Human epidermal growth factor receptor 2
ICI: Immune checkpoint inhibition
NACT: Neoadjuvant chemotherapy
NTA: Nano-tracking Analysis
OS: Overall survival
pCR: Pathological complete response
PD-1: Programmed death receptor
PD-L1: Programmed death receptor ligand-1
PD-L2: Programmed death receptor ligand-2
PFS: Progression-free survival
pNR: Pathological non-response
pPR: Pathological partial response
TNBC: Triple-negative breast cancer
