# Supplementary figures and images for: Programmed death receptor ligand-2 (PD-L2) bearing extracellular vesicles as a new biomarker to identify early triple-negative breast cancer patients at high risk for relapse
Source: J Cancer Res Clin Oncol. 2022 Apr 2;149(3):1159–74. doi: 10.1007/s00432-022-03980-9 (PMC9984327; doi:10.1007/s00432-022-03980-9)

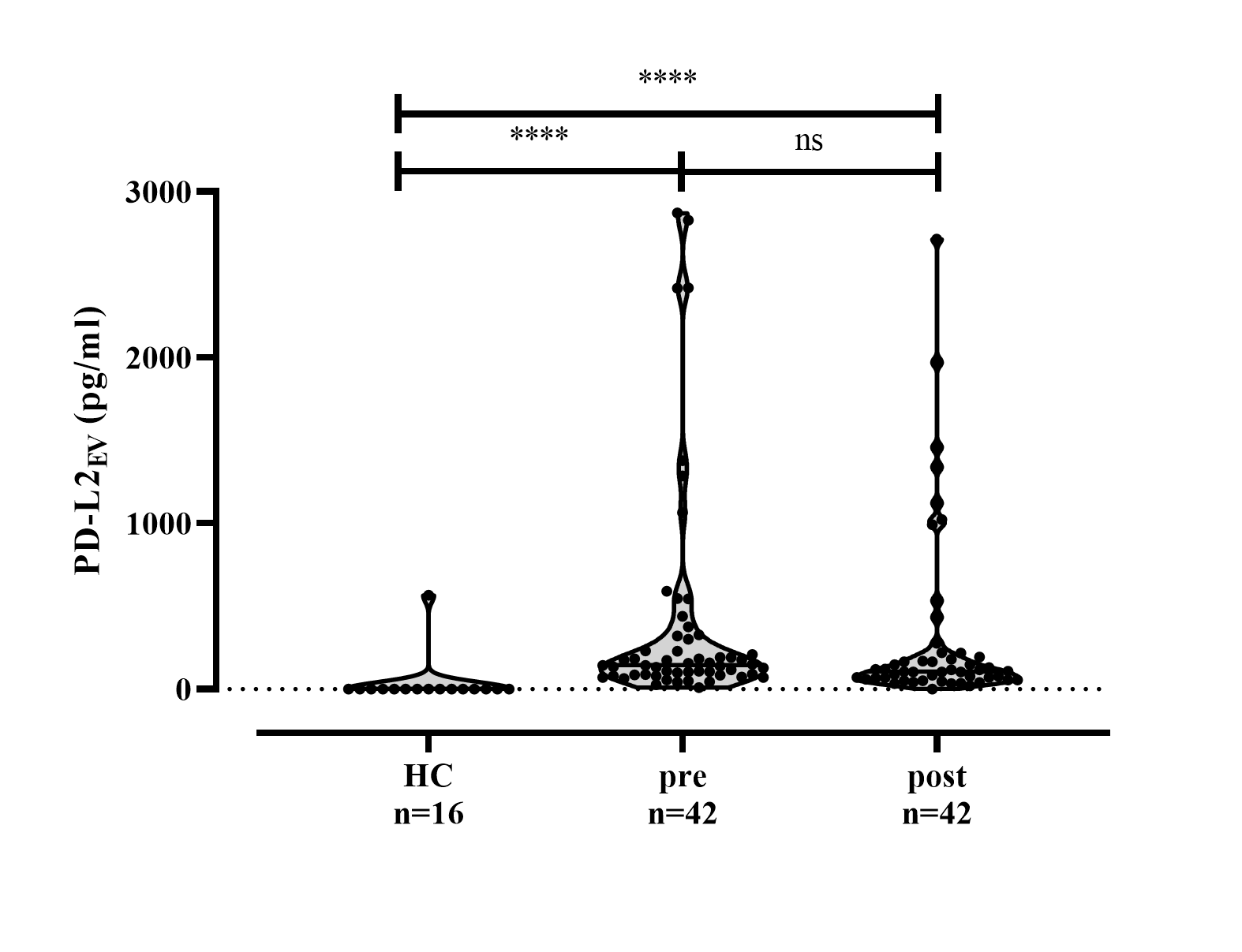

Supplement: Supplementary file 1 — Supplementary file1 (TIF 198 KB) [file 432_2022_3980_MOESM1_ESM.tif]
